# VA Video Connect for Clinical Care in Older Adults in a Rural State During the COVID-19 Pandemic: Cross-Sectional Study

**DOI:** 10.2196/21561

**Published:** 2020-09-30

**Authors:** Kalpana P Padala, Kerrie B Wilson, C Heath Gauss, Jessica D Stovall, Prasad R Padala

**Affiliations:** 1 Central Arkansas Veterans Healthcare System North Little Rock, AR United States; 2 University of Arkansas for Medical Sciences Little Rock, AR United States

**Keywords:** VA Video Connect, older adults, rural, COVID-19, veterans, telehealth, elderly, disparity, veteran affairs, capabillity, cross-sectional

## Abstract

**Background:**

The COVID-19 pandemic has accelerated the need for telehealth at home. Although the Department of Veterans Affairs is a leading provider of telehealth, disparities may exist in reaching older veterans living in rural areas. VA Video Connect (VVC) is a video conferencing app that enables veterans to connect with their health care provider via a secure and private session.

**Objective:**

The aim of this study was to examine the capability and willingness of older veterans to participate in a VVC visit during the COVID-19 pandemic.

**Methods:**

A cross-sectional study was conducted on older veterans (N=118) at the Central Arkansas Veterans Healthcare System. Participants were interviewed over the phone and responses to the following items were recorded: availability of internet, email, and an electronic device with a camera; veterans’ willingness to complete an appointment via a VVC visit; and availability of assistance from a caregiver for those who were unable to participate in a VVC visit alone.

**Results:**

Participants’ mean age was 72.6 (SD 8.3) years, 92% (n=108) were male, 69% (n=81) were Caucasian, 30% (n=35) were African Americans, and 36% (n=42) lived in a rural location. The majority reported having access to the internet (n=93, 77%) and email service (n=83, 70%), but only 56% (n=67) had a camera-equipped device. Overall, 53% (n=63) were willing and capable of participating in a VVC visit. The availability of internet access was significantly lower in rural compared to nonrural participants (*P*=.045) and in those with or less than a high school education compared to those who pursued higher education (*P*=.02). Willingness to participate in the VVC visit was significantly lower in rural compared to nonrural participants (*P*=.03). Of the participants who reported they were able and willing to partake in a VVC visit (n=54), 65% (n=35) opted for VVC and 35% (n=19) preferred a phone visit. In total, 77% (n=27) of the scheduled VVC visits were successful.

**Conclusions:**

Despite advances in technology, and willingness on the part of health care systems, there are some lingering issues with capability and willingness to participate in video telehealth visits, particularly among older adults residing in rural areas.

## Introduction

The COVID-19 pandemic has exposed many technological, ideological, and policy shortcomings in the telehealth transition. To ease policy burdens, the Centers for Medicare & Medicaid Services (CMS) has now expanded telehealth services for all Medicare beneficiaries while making virtual care reimbursable at the same rate as in-person visits, at least during the pandemic [1]. Governmental agencies have issued an emergency waiver suspending the requirement for compliance with the Health Insurance Portability and Accountability Act (HIPAA) and have noted that popular applications for video chats, such as Zoom video conference, Apple FaceTime, and Facebook Messenger video chat, which are not HIPAA compliant, may be used if necessary [2]. All major medical associations have urged health care providers to implement telehealth systems [3].

The Department of Veterans Affairs (VA) is a leader in telehealth and has been seeing an uptake in telehealth across the nation [4]. In 2018 alone, VA provided more than 2.29 million telehealth episodes of care through clinical video telehealth (CVT) services [5] to roughly 12% of the veteran population. Veterans can visit a community-based outpatient clinic (ie, VA satellite clinics often located in rural areas) close to their home and are connected to providers in the main medical centers via CVT. Of the veterans who received CVT, 45% lived in rural areas and may have otherwise had limited access to VA health care. However, less than 1% received care through a telehealth modality in their home or through other non-VA locations [6]. Additionally, the COVID-19 pandemic has encouraged staying home to reduce the potential exposure to infection, making CVTs less desirable. In 2018, VA unveiled VA Video Connect (VVC), a video conferencing app for veterans and VA providers as part of its *Anywhere to Anywhere* initiative [7]. VVC is a secure and private session that allows veterans to have real-time access to their VA care team from their home using the camera on their smartphone, computer, or tablet, and an email address to connect to staff. Although fairly well received, veterans in some studies expressed concerns about errors in their care, perceived providers paid less attention to them, and stated they were less comfortable speaking up and asking questions [8]. It is important to determine if there is a subgroup of veterans that is underserved by VVC.

Currently, there is a clear and collective national will to adapt technology to provide safe medical care, but it is very important to address the potential for creating a digital divide. Older veterans residing in rural areas may be particularly vulnerable to the digital divide. Rural residents account for a quarter of US veteran population and a third of the VA caseload [9,10]. Likewise, about 47% of veterans are over the age of 65 years and are much more likely to be living in rural areas compared with their younger counterparts [6,10]. Rural older veterans face the difficult choice of risking iatrogenic COVID-19 exposure during a clinician visit or postponing needed care [11]. We hypothesized that internet availability would be lower among rural older veterans compared to nonrural older veterans. Hence, the primary objective of this study was to examine the availability of internet access and the willingness of older adults in a rural state to participate in a VVC visit during the COVID-19 pandemic. The secondary objective was to examine the characteristics of veterans that make them less likely to be able or willing to participate in such visits. 

## Methods

### Sampling and Recruitment

A cross-sectional study was conducted over a 4-week period in the early part of the COVID-19 pandemic on older veterans who had appointments at the 6 clinics (5 geriatric clinics and 1 memory disorders clinic) of the Central Arkansas Veterans Healthcare System. All veterans scheduled for an appointment in these clinics during the study duration were invited to participate in the study.

The Institutional Review Board deemed this research to be exempt from review.

### Procedures

The study was introduced by the clinician and completed by a research assistant after obtaining verbal permission. All interviews were conducted over the phone. The questionnaire was developed by clinical researchers who routinely see patients and conduct clinical research in the target population. Some of the questions were adapted from previously published work by the team [12]. Participants were not compensated for their participation.

### Variables and Data Source

Demographic data, education, living situation, zip code, and comorbidities of the veterans were abstracted from the patient’s electronic medical records, which currently includes ICD-10 (International Statistical Classification of Diseases and Related Health Problems–10th revision) codes. Rurality was determined by searching the Office of Rural Health’s database by zip code.

A *My HealtheVet* account is a patient portal operated by VA through which veterans are able to see their laboratory results and communicate with their providers as needed. Whether or not veterans’ *My HealtheVet* accounts were linked to their electronic medical records was recorded.

### Information Collected During the Interview

During the interview, veterans were asked about their willingness to have their visit conducted via VVC instead of an in-person or CVT visit. As the VVC visit requires a participant to have an email account to receive the VVC link, veterans were asked if they had an electronic device with email access. If so, the type of device (eg, smartphone, tablet, or computer) and whether any of the devices had a camera attached or built into it were asked. They were also asked if they had internet access at home to complete the VVC visit and if they had ever used their *My HealtheVet* account. If veterans reported no to all the above questions, they were asked if they had a caregiver available to help them set up a VVC call. For those who reported caregiver availability, their relationship was noted.

### Information Collected Upon Completion of VVC Visits

All participants who were willing to and capable of using VVC appointments were asked if they had an upcoming appointment in the next 4 weeks. If they had an upcoming appointment, their preference for VVC vs phone call was recorded. A chart review was performed to ascertain if the VVC appointment was successfully completed and to capture any reasons noted in the chart for not completing a scheduled VVC appointment.

### Statistical Analysis

For demographic variables, descriptive statistics were obtained, including means, standard deviations, and percentages, as applicable. A veteran was considered capable of participating in a VVC visit if he or she had internet and email access and a device with a camera. To explore whether there was an association between internet access and willingness to participate in a VVC visit and rurality, a chi-square test or Fisher exact test was performed, as appropriate. A significance level of 5% was used. Exploratory analyses were also performed to test an association between internet access and educational status, *My HealtheVet* portal use or clinic visit type, and VVC willingness and/or capability using chi-square tests. The statistical analyses were conducted using SAS Enterprise Guide 7.15 (SAS Institute).

## Results

### Demographic and Descriptive Statistics

A total of 118 older adults participated in the cross-sectional interview during the COVID-19 pandemic stay-home period. All participants approached for the interview agreed to participate. Participants’ mean age was 72.6 (SD 8.3) years, 92% (n=108) were male, 68.6% (n=81) were Caucasian, and 29.7% (n=35) were African American (Table 1). The majority of the participants lived at home (n=116, 98%) and had a high school education/General Educational Development (GED) or some college/associate degree (n=79, 69%). Participants living in rural locations accounted for 36% (n=42) of the sample. In total, 40% (n=47) had their *My HealtheVet* account linked to their VA electronic medical records; of those, 81% (n=38) used their account. The most common chronic conditions included hypertension (n=96, 81%) and hyperlipidemia (n=54, 46%), followed by depression (n=38, 32%) and posttraumatic stress disorder (n=29, 25%); 26% (n=30) had either mild cognitive impairment or dementia (Table 1).

**Table 1:** Demographics and descriptive statistics (N=118).

Variable			Value	
**Age (years)**				
	Mean (SD)			72.6 (8.3)
	**Age category, n (%)**			
		≤64 years		16 (14)
		65-74 years		62 (52)
		≥75 years		40 (34)
Gender (male), n (%)			108 (92)	
**Race, n (%)**				
	Caucasian			81 (69)
	African American			35 (30)
	Native Hawaiian/Pacific Islander			2 (1)
**Education, n (%)**				
	Less than high school			1 (1)
	High school/GED^a^			31 (27)
	Some college/associate degree			48 (42)
	College/master’s/doctoral degree			34 (30)
**Living situation, n (%)**				
	Home			116 (98)
	Assisted living			0 (0)
	Nursing home			2 (2)
	Rural			42 (36)
***My HealtheVet* linked to CPRS^b^ (yes) , n (%)**			47 (40)	
	Used *My HealtheVet* account (n=47)			38 (81)
**Comorbidities, n (%)**				
	Dementia			14 (12)
	Mild cognitive impairment			16 (14)
	Depression			38 (32)
	Posttraumatic stress disorder			29 (25)
	Hypertension			96 (81)
	Hyperlipidemia			54 (46)
	Sensorineural hearing loss			24 (20)
	Macular degeneration			3 (3)
	Legally blind			0 (0)

^a^GED: General Educational Development.

^b^CPRS: Computerized Patient Record System.

### Baseline Characteristics of the Participants

In total, 93 out of 118 participants (77%) had internet access, and 83 (70%) participants had email access; however, only 67 (56%) participants had a device with a camera (Table 2). The majority used a desktop computer (44/83, 53%) or a smartphone (28/83, 34%) to access their email. Among those with a device, 67 out of 83 participants (81%) had a device with a camera. Of the 118 participants, 66 (56%) were capable of participating in a VVC visit and 69 (58%) expressed willingness. Approximately half the veterans (n=63, 53%) were both capable and willing to participate in an appointment via VVC. Almost all of those capable were willing to participate in the appointment via VVC (63/66, 95%). Among those not capable, 6 out of 52 participants (12%) were willing to participate in the appointment via VVC.

**Table 2:** Details on capability and willingness to participate in a VA Video Connect (VVC) appointment (N=118).

Variable			Participants, n (%)
Availability of internet access			91 (77)
Availability of email access			83 (70)
Availability of a device with a camera			67 (56)
Capable of participating in VVC appointments^a^			66 (56)
Willingness to participate in VVC appointments			69 (58)
Capable and willing to participate in VVC appointments			63 (53)
**Type of device available (n=83)**			
	Smartphone	28 (34)	
	Tablet	6 (7)	
	Desktop computer	44 (53)	
	All above devices	5 (6)	
Availability of a caregiver to help set up the VVC appointment (n=40)			23 (58)
**Relationship of the caregiver (n=23)**			
	Spouse	13 (57)	
	Sibling	2 (9)	
	Child	7 (30)	
	Other	1 (4)	

^a^Older adults with availability of internet access, email, and a device with camera were considered capable to do the VVC appointments.

### Information Collected During the Interview

#### Primary Analyses

The proportion of participants who had access to the internet was significantly lower in rural veterans compared to nonrural ones (*P*=.045). The proportion of rural participants who were willing to participate in a clinic visit via VVC was significantly lower compared to nonrural participants (*P*=.03). The differences in availability of various resources and capability and willingness among rural and urban veterans are depicted in Table 3.

**Table 3:** Differences in the availability of various resources and capability and willingness among rural and urban veterans.

Variable	Participants		
	Rural, n (%)	Urban, n (%)	*P* value
Internet	28 (67)	63 (83)	.04^a^
Email	26 (62)	57 (75)	.14
Device with a camera	20 (47)	47 (82)	.55
Capable^b^	20 (48)	46 (61)	.18
Willing	19 (45)	50 (66)	.03^a^
Capable and willing^c^	18 (43)	45 (59)	.09

^a^*P* value <.05 is clinically significant.

^b^Capable: a veteran was considered capable of participating in a VVC visit if he or she had internet access and also had a smartphone, tablet, or computer that had both email access and a camera.

^c^Capable and willing: a veteran capable and willing to participate in a VVC visit.

#### Secondary and Exploratory Analyses

The proportion of participants who had internet access was significantly lower in those with high school or less education compared to those with more than high school education (*P*=.02). The proportion of participants who had access to the internet and email service was significantly lower for those who had an appointment at the memory disorders clinic (*P*=.01) compared to those who had an appointment at the other geriatric clinics (*P*=.01). The proportion of veterans who had their *My HealtheVet* account linked to their electronic medical records expressed significantly higher capability in participating in a VVC visit (*P*=.001), willingness to participate in a VVC visit (*P*=.001), and capability and willingness to participate in a VVC visit (*P*=.003), compared to those who did not have their *My HealtheVet* account linked to their electronic medical records.

### Attempts and Completion of VVC Visits

Of the 63 VVC-capable and willing veterans, 54 had upcoming visits in the aforementioned clinics within 4 weeks from the time of their interviews. Of these 54 veterans, 19 (35%) preferred their appointment over the phone and 35 (65%) preferred a VVC visit. A total of 35 VVC appointments were scheduled during the study period. Of these, 27 (77%) were successfully completed. Of the completed VVC appointments, 13 (48%) received assistance from a relative or caregiver for the appointment. In total, 6 veterans could not complete their VVC appointments; 4 had internet connectivity issues and 2 were no-shows for their appointments.

## Discussion

### Principal Findings

This study describes the feasibility of conducting VVC visits in a rural state. The findings from this study are encouraging, with 77% of older adults having access to the internet and 70% having email service. These findings are similar to other studies that found increasing internet use in older veterans and willingness to participate in video programs for health [11]. It is troubling, however, to see that the historically described disparities in internet and email access among rural veterans is still prevalent, albeit at a lower frequency. Education appears to have a stronger influence on internet access, although willingness to participate in a VVC visit was more strongly influenced by rurality than education. These findings suggest that older veterans with a high school or less education residing in rural area—the very consumers that need the VVC services the most—may require extra attention. The Federal Communications Commission has taken key steps to bring broadband to rural America, such as the $20-billion investment in the *Rural Digital Opportunity Fund* and $1.4-billion allocation for the *Connect America Fund Phase II Reverse Auction* to expand broadband to more than 700,000 rural locations in 45 states [13]. Future measures are needed to make internet services affordable to rural residents.

The biggest hurdle for VVC capability in this sample was the availability of a camera-enabled device; only 56% of the participants had this. It is interesting to note that almost all of those who were capable of participating in a VVC appointment were willing to do so (95%). This suggests that providing video-enabled devices could improve uptake of VVCs. VA has been giving tablets to select groups of veterans for telehealth since 2016. Tablet recipients (N=604) reported that the care they received via video consultations was equivalent to in-person visits [14]. However, in the same study, 20% of the participants did not use the tablets, and one third, those with technological difficulties and/or multiple comorbidities, preferred in-person visits over remote visits [14]. However, the survey respondents in this study were younger (mean age 56.0, SE 0.20 years) than those of the current study [14]. In our study, only 12% of those that did not have VVC capability were willing to engage in VVCs, suggesting that providing tablets, although an important first step, may not solve problems for all veterans. Nevertheless, such selected patients could be targeted for tablet provision after ensuring that an internet connection is in place.

The participants that had an appointment at the memory disorders clinic had significantly lower access to internet or email services compared to those enrolled in other clinics. This difference could be due to a cohort effect and may be ameliorated by the presence of caregivers. Home-based video programs for dementia care are pioneered by groups such as Dr Lauren Moo’s team at the Bedford Geriatric Research, Education and Clinical Center, a center of excellence within VA focused on enhancing geriatric care [15]. The availability of such home-based video programs for dementia care has expanded exponentially across the nation [15]. With VA’s growing emphasis on caregiver services, telehealth options for veterans with dementia could grow exponentially.

The strong association between *My HealtheVet* portal use and VVC willingness and capability is very encouraging as the use of this portal by veterans could be a proxy for VVC capability and/or willingness. This finding, if proven in larger studies, may help assess generalizability; a clinician could then simply check if a veteran is currently using the portal before making the decision to offer a VVC visit.

The results of this study should be considered in the context of several limitations. The study population consisted of US veterans, of mostly male gender, and from a single geographic area, and thus, the patient-reported responses in our results may not be generalizable to other population groups, women, or other areas that use different telehealth equipment, workflows, and processes of care. Despite these limitations, our study can be characterized by many strengths, including the systematic collection of actionable information from a group of veterans who are older and at high risk of being left out in the current digital transformation.

### Conclusion

Despite advances in technology, and willingness on the part of health care systems, there are some lingering issues relating to capability and willingness to participate in video telehealth visits particularly among older adults residing in rural areas. Policy efforts to bring broadband internet access to rural residents are the necessary first steps in addressing the digital divide. Targeted educational efforts are needed to train those residing in rural areas. The distribution of video-capable devices needs to be augmented by the provision of high-speed internet and age-friendly technical assistance.

## Abbreviations

CMS: Centers for Medicare and Medicaid Services
HIPAA: Health Insurance Portability and Accountability Act
CVT: clinical video telehealth
VA: Department of Veterans Affairs
VVC: VA Video Connect
